# Automatic detection and visualisation of MEG ripple oscillations in epilepsy

**DOI:** 10.1016/j.nicl.2017.06.024

**Published:** 2017-06-17

**Authors:** Nicole van Klink, Frank van Rosmalen, Jukka Nenonen, Sergey Burnos, Liisa Helle, Samu Taulu, Paul Lawrence Furlong, Maeike Zijlmans, Arjan Hillebrand

**Affiliations:** a Brain Center Rudolf Magnus, Dept. Neurology and Neurosurgery, UMC Utrecht, Utrecht, The Netherlands; b MIRA Institute for Biomedical Technology and Technical Medicine, Twente University, Enschede, The Netherlands; c Elekta Oy, Helsinki, Finland; d Neurosurgery Department, University Hospital Zurich, Zurich, Switzerland; e Department of Neuroscience and Biomedical Engineering, Aalto University, Espoo, Finland; f Department of Physics, University of Washington, USA; g Institute for Learning and Brain Sciences, University of Washington, USA; h Wellcome Trust Laboratory for MEG Studies, Aston Brain Centre, Aston University, Birmingham, United Kingdom; i SEIN – Stichting Epilepsie Instellingen Nederland, Heemstede, The Netherlands; j Department of Clinical Neurophysiology and Magnetoencephalography Center, VU University Medical Center, Amsterdam, The Netherlands

**Keywords:** Magnetoencephalography, Epilepsy, Beamformer, Virtual sensors, Automatic detection, High frequency oscillations

## Abstract

High frequency oscillations (HFOs, 80–500 Hz) in invasive EEG are a biomarker for the epileptic focus. Ripples (80–250 Hz) have also been identified in non-invasive MEG, yet detection is impeded by noise, their low occurrence rates, and the workload of visual analysis. We propose a method that identifies ripples in MEG through noise reduction, beamforming and automatic detection with minimal user effort. We analysed 15 min of presurgical resting-state interictal MEG data of 25 patients with epilepsy. The MEG signal-to-noise was improved by using a cross-validation signal space separation method, and by calculating ~ 2400 beamformer-based virtual sensors in the grey matter. Ripples in these sensors were automatically detected by an algorithm optimized for MEG. A small subset of the identified ripples was visually checked. Ripple locations were compared with MEG spike dipole locations and the resection area if available. Running the automatic detection algorithm resulted in on average 905 ripples per patient, of which on average 148 ripples were visually reviewed. Reviewing took approximately 5 min per patient, and identified ripples in 16 out of 25 patients. In 14 patients the ripple locations showed good or moderate concordance with the MEG spikes. For six out of eight patients who had surgery, the ripple locations showed concordance with the resection area: 4/5 with good outcome and 2/3 with poor outcome. Automatic ripple detection in beamformer-based virtual sensors is a feasible non-invasive tool for the identification of ripples in MEG. Our method requires minimal user effort and is easily applicable in a clinical setting.

## Highlights

• Cross-validation signal space separation and beamformer increase the SNR in MEG.
• Automatic detection of MEG ripples in the time domain is feasible.
• Our method identifies ripples with minimal user effort and is clinically applicable.
• Automatically detected ripples are concordant with MEG spikes in 14/16 patients.
• Automatically detected ripples are concordant with resection area in 6/8 patients.

## 1 Introduction

All investigations in the workup for epilepsy surgery aim to identify the epileptogenic zone sensitively and specifically. The trade-off between sensitivity and specificity often involves the invasiveness of the investigation. Interictal epileptiform discharges, also called spikes, in electroencephalography (EEG), electrocorticography (ECoG) and magnetoencephalography (MEG) are often used to estimate the location of the epileptogenic zone, but spikes might not be very specific (Sugano et al., 2007, Wennberg et al., 2011). High frequency oscillations (HFOs, 80–500 Hz) are electrophysiological transients that are used as biomarkers for the epileptogenic zone in ECoG, and show a high sensitivity and specificity (Fujiwara et al., 2012, Jacobs et al., 2010, van't Klooster et al., 2015). The use of HFOs as a biomarker in non-invasive investigations is a topic of current research. Ripples (80–250 Hz) have been found in both EEG and MEG (Andrade-Valenca et al., 2011, Kobayashi et al., 2015, Van Klink et al., 2016a, Van Klink et al., 2016c). A specific and sensitive non-invasive biomarker would reduce the need for invasive investigations.

MEG is a promising recording technique for ripple analysis, because of its generally higher spatial resolution than clinical EEG. Analysis of ripples in MEG is a recent development. Few MEG studies have analysed high gamma or ripples in patients with epilepsy, either by looking at the spectral content (Guggisberg et al., 2008, Miao et al., 2014, Tang et al., 2015, Tenney et al., 2014, Xiang et al., 2015), or by searching for short lasting oscillations that stand out from the baseline (Papadelis et al., 2016, Van Klink et al., 2016a, Von Ellenrieder et al., 2016).

The large number of sensors in modern whole-head MEG systems is an advantage for localization, but makes visual analysis of ripples very time consuming. Automatic detection algorithms for invasive ripples have been developed, but direct application to MEG signals is difficult due to differences in signal characteristics. A recent study (Von Ellenrieder et al., 2016) used a detection algorithm to find ripples in MEG based on an increase in root mean square amplitude in 10 narrow frequency bands between 40 and 160 Hz. After rejection of possible artefacts and visual validation by two reviewers, ripples were identified in 8 out of 17 patients. This algorithm was developed to detect ripples with a high sensitivity. Another algorithm, developed by Burnos and colleagues (Burnos et al., 2014, Burnos et al., 2016), identifies possible ripples by using the Stockwell entropy (Stockwell et al., 1996) of the signal and detects ripples based on the presence of a high frequency component with well-defined characteristics in the time-frequency spectrum. This algorithm was designed to detect ripples with a high specificity for the seizure onset zone.

The low amplitude of the ripples, combined with high amplitude background noise, result in a low signal-to-noise ratio (SNR) and mean that it can be hard to (automatically) distinguish ripples from the baseline. In a previous study we have shown that the use of beamformer virtual sensors can increase the signal-to-noise ratio, and show ripples that were not visible in the physical sensors (Van Klink et al., 2016a). These ripples were marked visually for 70 virtual sensors placed in a priori defined areas of interest. Covering the whole head with virtual sensors would increase the sensitivity, but at the same time would hugely increase the number of channels, rendering visual analysis impractical.

The aim of this study was to generate beamformer virtual sensors throughout the cortex to increase the chance of finding ripples, and to detect these ripples with an automatic detection algorithm with as little manual reviewing as possible. To enable automatic detection, we further increased the SNR by pre-processing the data with the extended signal space separation (xSSS) method, which combines efficient interference elimination and reduction of sensor noise (manuscript in preparation). We adapted the ripple detector algorithm developed by Burnos et al., 2014 to work with our MEG virtual sensor data. With this detector it was possible to automatically analyse the approximately 2400 beamformer virtual sensors for the presence of ripples, showing that the approach would be applicable in a clinical setting. We compared the identified ripple locations to the clinical information of each patient in order to determine the validity of the approach.

## 2 Methods

### 2.1 Patients

Patients with refractory epilepsy in the presurgical workup for epilepsy surgery at the University Medical Centre Utrecht, who had an MEG registration in 2012 or 2013 at the VU University Medical Centre in Amsterdam, were included. Patients without epileptic spikes in the MEG, according to the clinical report, were excluded, since patients with spikes have a higher chance of showing ripples (Melani et al., 2013). Also MEG recordings with extensive high frequency artefacts were excluded. We determined the resected brain area in patients who had undergone surgery based on post-surgical MRI (if available) or based on a description of the surgery. Patients were considered seizure free if they had an Engel score of 1 at the longest available follow up. All patients or caretakers gave written informed consent for use of their data for research.

### 2.2 MEG data acquisition

MEG recordings were performed with a 306-channel whole head Elekta Neuromag® system (Elekta Oy, Helsinki, Finland) in a magnetically shielded room (VacuumSchmelze GmbH, Hanau, Germany). The system consists of 102 sensor units, each with two gradiometers and one magnetometer. Four or five head localization coils continuously recorded the position of the head in the MEG helmet. The data were recorded with a 1250 Hz sampling frequency, a low-pass anti-aliasing filter of 410 Hz and a high-pass filter of 0.1 Hz. Recordings were made with closed eyes, and in a supine position, to minimize head movement. A fifteen-minute resting-state interictal recording was used for analysis. Other recordings included a motor task and somatosensory stimulation, but these data were not used in this study. The position of the head localization coils and the shape of the scalp were digitized using a 3D digitizer (Fastrak, Polhemus, Colchester, VT, USA).

### 2.3 Anatomical MRI

Each MEG recording was co-registered with a T1-weighted structural magnetic resonance image (MRI) of the patient with surface matching software developed by one of the authors (AH). This resulted in a co-registration error of approximately 4 mm (Whalen et al., 2008). A single sphere, which fitted best to the outline of the scalp, was used as volume conductor model. This model was used for the beamformer analysis described below.

**Fig. 1:**
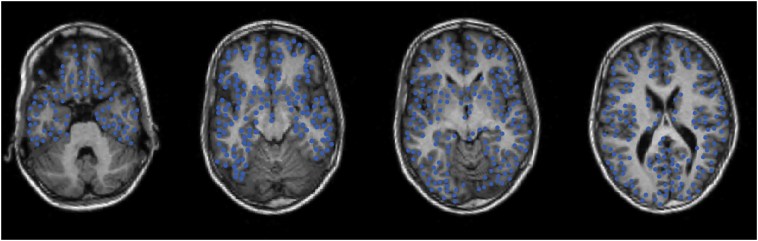
Locations of beamformer virtual sensors for 4 different axial slices in patient 22. All grey matter voxels were segmented from the MRI and down sampled to a minimum inter-sensor distance of 5 mm. Cerebellar grey matter voxels were excluded, but deep structures like the hippocampus and interhemispheric grey matter were maintained.

We used the same T1 MRI to reconstruct virtual sensors in the grey matter. This was done by segmenting the grey matter in SPM12 in Matlab (version 8.5.0; Mathworks Inc., Natick, MA, USA), down sampling the grey matter voxels to get a minimum inter-sensor distance of 5 mm, and excluding all voxels below the nose. Cerebellar grey matter voxels were excluded, but deep structures like the hippocampus and interhemispheric grey matter were maintained. The remaining voxels were used as virtual sensor locations. The coverage of virtual sensors was visually checked for each patient. Each patient had between 2060 and 2788 virtual sensor locations (average 2421, Fig. 1).

### 2.4 Data processing

We removed the signal from the head localization coils with a band-stop filter and applied the new cross-validation signal space separation (xSSS) method implemented in a research software module (Elekta MaxFilter version 3.0, not commercially available). Compared to the spatial SSS (Taulu and Kajola, 2005) and spatiotemporal tSSS (Taulu and Simola, 2006), the xSSS method has two important novelties: cross-validation for extracting and suppressing uncorrelated channel artefacts and noise, as well as covariance-based regularization of the SSS reconstruction for reducing the sensor noise. Details of the xSSS pre-processing are described in Appendix A.

We used a scalar beamformer similar to Synthetic Aperture Magnetometry (Robinson and Vrba, 1999) that is implemented in a research software module (Elekta Beamformer version 2.2, not commercially available). The 80 Hz high-pass-filtered, pre-processed signal was used for data covariance, and the first 10 s of the unfiltered pre-processed signal were used to estimate noise covariance. Both magnetometer and gradiometer data were used to calculate the beamformer solution, so that the relative advantages of the two sensor-types are combined (i.e. magnetometers for deeper sources; gradiometers with higher SNR for superficial sources). Normalized beamformer weights were calculated and used to reconstruct time series for the virtual sensor locations (Cheyne et al., 2007, Hillebrand and Barnes, 2005, Hillebrand et al., 2005).

### 2.5 Ripple detection

**Fig. 2:**
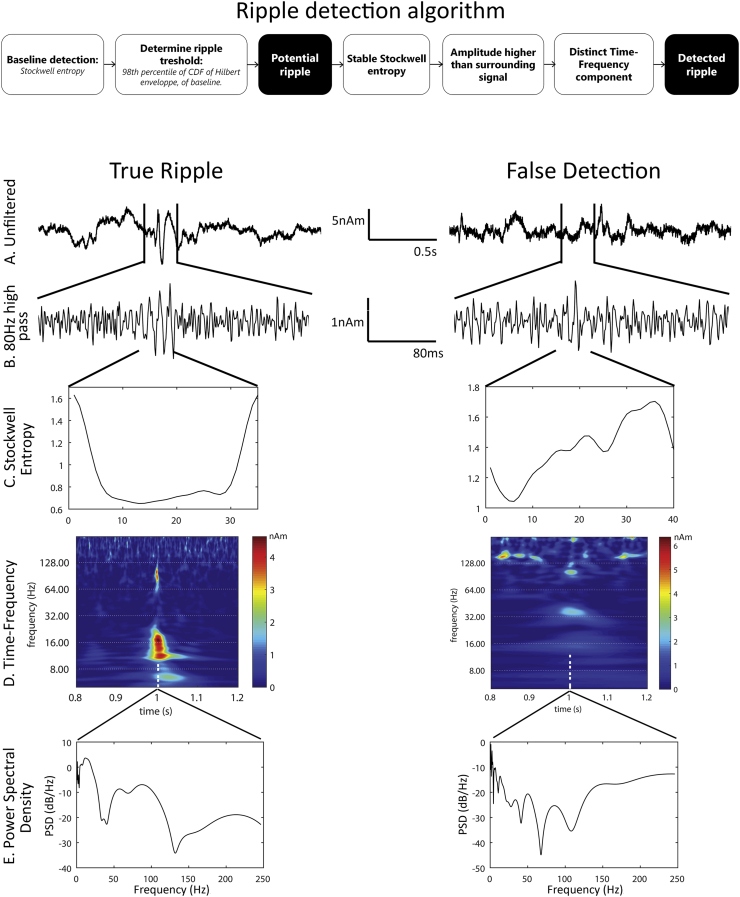
Schematic overview of automatic ripple detection algorithm, with examples of a true ripple (left) and an event that is not in the final output of the detector (right), because the entropy is not stable over the length of the event. A) Unfiltered virtual sensor signal. B) 80 Hz high-pass filtered signal, showing the true ripple (left) and false detection (right). C) Stockwell entropy over the length of the event is stable for the true ripple (left), and irregular for the false detection (right). D) Time-frequency decomposition shows a high frequency component for the true ripple (~ 100 Hz) and the spike that can be seen in part A (12–20 Hz, left), and less distinct components and high frequency artefacts for the false detection (right). E) The power spectral density (PSD) also shows the high frequency component in the true ripple (60–100 Hz, left), and the irregular high frequency activity for the false detection (right).

Ripples were automatically detected in all virtual sensors by an adapted version of the HFO detector developed by Burnos et al., 2014, Burnos et al., 2016. The original detector has previously been optimized for use on intracranial grid and depth electrode signals, which have a higher SNR than non-invasive MEG signals. We adapted the parameters of the detector and added extra requirements for true ripples to deal with the increased noise levels. The detector filtered all channels with an elliptic band pass filter between 70 and 253 Hz (− 3 dB points) with a stop band attenuation of 60 dB on both sides, and a band pass attenuation of 0.5 dB. The algorithm was applied on filtered individual channels and has a two-step approach: first a baseline was identified by computing the Stockwell entropy for 120 random one second epochs; samples with entropy higher than the threshold (0.85 ∗ maximum entropy) were considered as baseline. In the second step the ripples were identified. An envelope for all baseline segments was calculated with the Hilbert transform, a cumulative distribution function (CDF) of all segments was constructed, and the 98th percentile of this CDF was used as a threshold for potential ripples for that channel. When the Hilbert envelope of a channel exceeded this channel threshold for at least 20 ms, a potential ripple was found. A true ripple was defined when for a potential ripple a) the Stockwell entropy during the event was stable; the maximum entropy was smaller than 125% of the minimum entropy, excluding the first and last sample, b) the absolute amplitude was higher than the absolute amplitude plus one standard deviation of 1000 samples before and 1000 samples after the potential ripple, and c) a distinct component was present in the time-frequency spectrum between 40 and 250 Hz, detected by a peak above 40 Hz preceded by a trough in the power spectral density (PSD, Fig. 2).

**Fig. 3:**
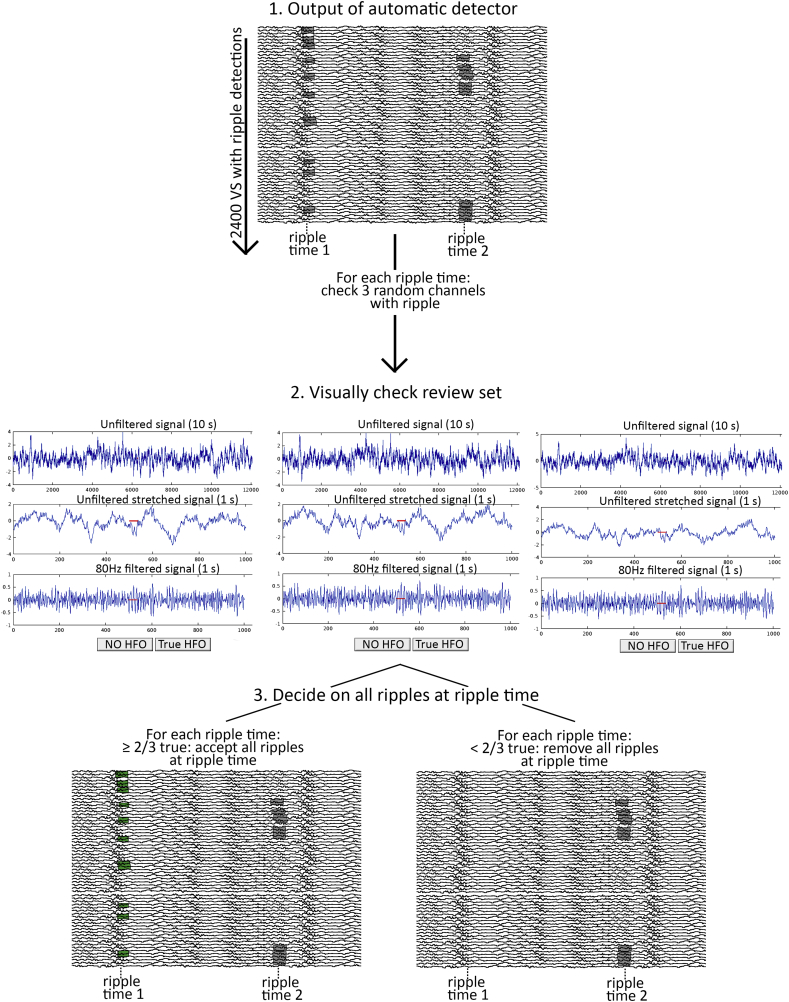
Schematic overview of the review process. The automatic detector has detected ripples in all ∼ 2400 virtual sensor channels. All moments in time where at least one ripple was detected (ripple-times) were extracted, and a review set was comprised by a maximum of three randomly chosen virtual sensors with ripples at each ripple-time. The reviewer was presented a 10 s trace of the unfiltered virtual sensor at the time of the ripple, a 1 s trace of the unfiltered virtual sensor and a 1 s trace of the 80 Hz high pass filtered virtual sensor, with the marked event in all traces. The reviewer determined if the automatically detected ripple was true or not. If > 2/3 of the reviewed ripples at a ripple-time were considered true, all ripples at that ripple-time in all channels were considered true, also the ripples that were not included in the review set.

**Fig. 4:**
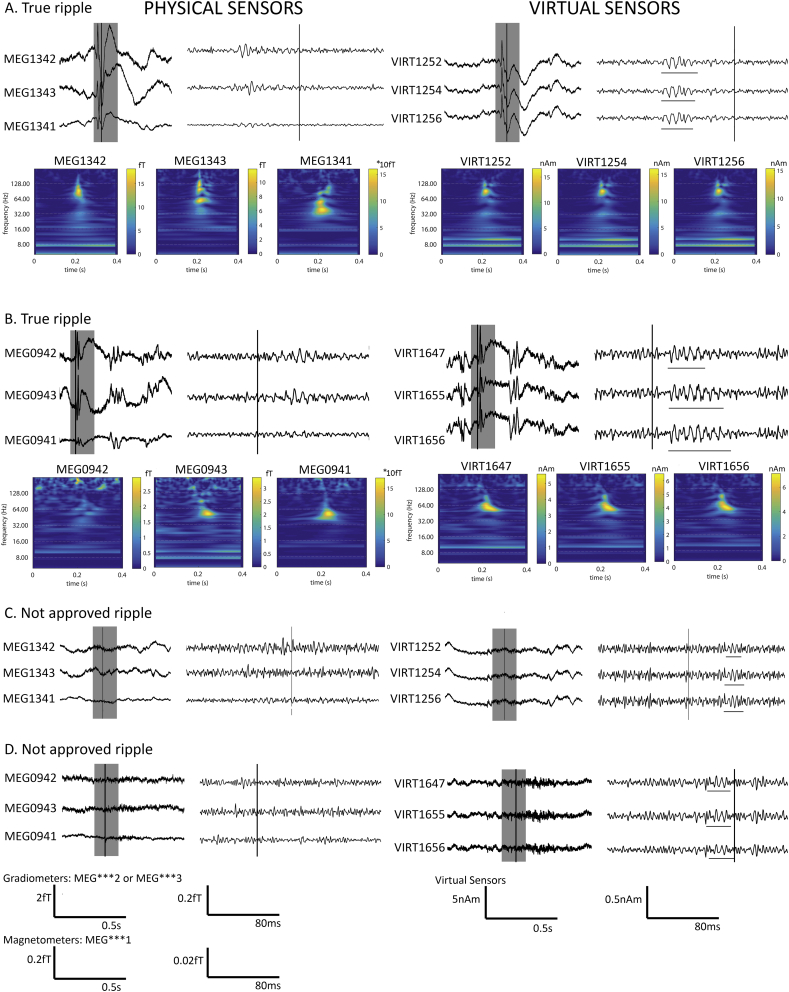
Examples of ripples that were approved (A + B) and not approved (C + D) during the visual check. On the left side we show the physical sensors after xSSS preprocessing closest to the virtual sensors that are shown on the right. The left part of each sensor set shows unfiltered data. The grey area is 80 Hz high pass filtered and shown on the right. Vertical lines indicate the same moment in time. In part A and B the true ripples are underlined, and a time frequency spectrum of each signal is shown below. Some sign of the ripple can be found in the physical channels, but only the virtual channels show a clear ripple. In part C and D the falsely marked ripples by the detector are underlined. These were discarded by the reviewer and not used for further analysis.

As automatic ripple detectors have the tendency to include a large number of false positives, we checked the performance of the detector in each patient by visually reviewing a selective set of detected (‘true’) ripples. All moments in time that at least one ripple was detected (ripple-times) were extracted, and the review set was comprised by a maximum of three randomly chosen virtual sensors with ripples at each ripple-time. The reviewer was presented a 10 s trace of the unfiltered virtual sensor at the time of the ripple, a 1 s trace of the unfiltered virtual sensor, and a 1 s trace of 80 Hz high pass filtered virtual sensor, with the marked event in all traces, in a custom-made graphical user interface. The reviewer determined if the automatically detected ripple was true or not. If more than half of the reviewed ripples at a ripple-time were considered true, all ripples at that ripple-time in all channels were considered true, also the ripples that were not included in the review set. As the review set consisted of maximum three ripples at a certain ripple-time, all ripples at that ripple-time were considered true if > 66% of the ripples in the review set were considered true (Fig. 3). This strategy minimized the number of ripples to be reviewed, while all ripple-times were evaluated. Potentially true ripples at the same time as artefact detections at other channels could be excluded with this approach. The reviewer was blinded for the clinical information and for the location of the channel that was reviewed. The location of the ripples in the analysis is the location of the virtual sensors in which ripples were detected. We did not systematically review the raw MEG data at the same time-points, because in an earlier study we found that at 78% of the ripple-times, the raw MEG only showed noise (Van Klink et al., 2016a). We did review the unfiltered virtual sensor data to decrease the chance of marking artefacts. Fig. 4 shows examples of events that were considered true ripples, and events that were considered artefacts, together with the physical sensor channels.

### 2.6 Spike dipole analysis

The primary, non-propagated, and therefore clinically most important epileptic spikes in the physical sensor channels were marked and evaluated for a clinical report by a team of clinicians, MEG/EEG technicians and physicists. These primary spikes were localized with a dipole fit at every sample from half-way of the flank preceding the top, to the top of the spike, with a single moving equivalent current dipole (using the Elekta Source Modelling software version 5.5). The locations of the fitted dipoles were used to compare with the locations of the ripples.

### 2.7 Analysis

The results of the ripples after automatic detection and review were visualized on axial slices of the patient's MRI, and in a 3D figure. The concordance between the area(s) with ripples and the area(s) with spikes in the MEG was assessed visually and was classified as good (+) if all ripples were located in the same lobe as the spike dipoles, moderate (=) if any ripple was located in the same lobe as the spike dipoles, and bad (−) for discordance. A similar classification strategy was used to assess the concordance between the area with ripples and the resected brain area for those patients who had undergone surgery. Concordance was good (+) if > 50% of the ripple locations were included in the resection, at lobar level, moderate (=) if < 50% ripple locations were included in the resection, and bad (−) for discordance. We classified the concordance between the MEG spike dipole locations and the resected brain area by using the same criteria as for ripples.

Twelve patients (14–25) had already been included in a previous study in which we visually marked ripples in a predefined area of interest using the same MEG recordings (Van Klink et al., 2016a). Here, we were therefore able to compare the number of automatically identified ripple-times to the number of visually marked ripple-times in these patients.

Statistical analyses were performed using IBM SPSS Statistics 23 (IBM Corp., Armonk, NY, USA); a *p*-value < 0.05 was considered significant.

## 3 Results

### 3.1 Patients

Fifty-eight patients had an MEG recording in 2012 or 2013, of whom 32 did not show epileptic spikes in the clinical analysis. The MEG of one patient showed such artefacts that the patient had to be excluded from the analysis. The other 25 patients were included: they had a mean age of 12 years (range: 4–29) and 19 were male. Fifteen patients had undergone epilepsy surgery, for which the resection area was determined based on all available presurgical investigations, including MEG spikes (Table 1). Ten patients were seizure free after surgery (Engel 1 outcome). The average follow up time for all patients was 2.2 years (range: 0.5–4 years).

**Table 1:** Patient characteristics, showing the location of MEG spikes, interictal EEG abnormalities, ictal EEG onset, PET abnormalities, SPECT abnormalities, pathology and/or MRI findings, and surgery with Engel outcome and duration of follow-up.

Pt #	Gender/age	MEG spikes	Interictal EEG abnormalities	Ictal EEG onset	PET	SPECT	Pathology/MRI	Surgery (outcome)
1	M/21	L/frontal	L frontal	NA	L temporal	NA	MRI no abnormalities	No surgery
2	M/8	R/frontal	R centrotemporal	R Frontocentroparietal	NA	NA	Multiple cortical tubers	Subtotal resection R frontal tuber (1A, 3y)
3	F/14	Bilateral frontal	Frontal R > L	R frontal	R frontal	NA	MRI no abnormalities	No surgery
4	M/25	R/central	R parietocentral	R parietocentral	NA	NA	Ganglioglioma WHO I R postcentral	Lesionectomy R postcentral (1A, 3y)
5	F/28	R temporoparietal	Bilateral frontal	R frontal	NA	NA	Ganglioglioma WHO I R frontal	Lesionectomy R frontal (1A, 4y)
6	M/5	Bilateral centrotemporal	R centrotemporal	NA	No abnormalities	NA	MRI no abnormalities	No surgery
7	M/12	Bilateral fontal parasagittal	Bilateral frontal	Bilateral frontal	No abnormalities	NA	MRI no abnormalities	No surgery
8	M/5	L frontal + L mesiotemporal	L frontal	L Frontal/frontotemporal	NA	NA	Cyst L frontal	L frontal disconnection (3A, 3y)
9	M/4	R occipital	R frontal + L parietal	R parietal with fast spread to frontal	R parieto-occipital	NA	FCD ILAE IC	R parietooccipital disconnection (3A, 3y)
10	F/5	Diffuse/multifocal	R frontocentral	NA	NA	NA	Porencephalic cyst R and tissue degeneration of basal nuclei	R hemisferectomy (1A, 2y)
11	M/14	L frontal + temporooccipital	L parietal	L posterior temporal	L temporoparietal	NA	Ganglioglioma L anterior basotemporal, WHO I, with associated FCD, ILAE IIIB	Lesionectomy L temporo-occipital basal (2A, 3y)
12	M/4	R frontal + widespread	Multifocal: R frontolateral, R temporal, L temporal	No lateralisation or localisation	NA	Multifocal (L TP, L T, L PO, R P)	Multiple cortical tubers	No surgery
13	M/13	Bilateral frontal and temporal	Multifocal: R frontolateral, R occipital, L frontolateral	Multifocal, most prominent R frontolateral	NA	R frontal	Multiple cortical tubers	Lesionectomy R frontal and temporal (4A, 3y)
14	M/7	L/temporal	Possible frontal focus, probably R	No lateralisation or localisation	No abnormalities	NA	MRI no abnormalities	No surgery
15	M/15	R/temporal basal	R fronto tempero basal	R, not localizing	NA	R temporal	Multiple cortical tubers, decreased grey and white matter differentiation R temporal	R temporolobectomy with amygdalohippocampectomy (1A, 6md)
16	M/16	R/temporal basal	R anterior temporal	R temporal	R temporal	R temporal	MRI no abnormalities	R temporolobectomy with amygdalohippocampectomy (1D, 1y)
17	F/16	L/temporoparietal	L fronto temporo basal	R or L in different seizures.	No abnormalities	L temporal	Minimal white matter malformations R frontal	No surgery
18	F/17	R/frontocentral	Frontocentral midline, probably more L	Bilateral frontocentral	R central	NA	MRI no abnormalities	No surgery
19	M/10	Bilateral frontal	Bilateral frontal and generalized	NA	NA	NA	Arachnoidal cyst L temporal	No surgery
20	M/6	L/temporal posterior	L temporal, more posterior	L posterior temporal	NA	NA	Multiple cortical tubers + SEGA R near intraventricular foramen	Resection of growing SEGA 3rd ventricle + tuber R frontal (4B, 1.5y)
21	M/6	R/parietal	R central paramedian	R central paramedian	R frontal or parieto-occipital	NA	Multifocal gliosis, R > L	No surgery
22	M/12	R/temporal posterior	R hemisphere, most temporal	R centrotemporal	R temporo-parieto-occipital	R temporo-parietal	MTS R, Wyler IV	R temporolobectomy with amygdalohippocampectomy and lesionectomy R posterior parietal (1A, 1y)
23	M/14	R/temporal	R posterior temporal	R posterior temporal	R posterior temporal	NA	FCD ILAE IIA, R occipitotemporobasal	R occipitotemporobasal resection (1A, 2y)
24	M/12	R/frontocentral	R frontal and centroparietal	R temporal diffuse	R anterior temporal	NA	Ventricular cyst R frontal + MTS R, Wyler 2	R anterior temporolobectomy with amygdalohippocampectomy (1C, 1.5y)
25	F/29	L/parietotemporal	No clear interictal epileptiform discharges	L frontal and midline	NA	NA	Arteriovenous fistula L parietooccipital	Lesionectomy of fistula, unable to resect seizure focus due to speech arrest (1D, 1.5y)

*M: male, F: female, L: left, R: right, SEGA: subependymal giant cell astrocytoma, FCD: focal cortical dysplasia, MTS: mesiotemporal sclerosis, WHO: world health organization classification for tumors, ILAE: international league against epilepsy classification for focal cortical dysplasia, NA: not available*.

### 3.2 MEG pre-processing

The previous study (Van Klink et al., 2016a) utilized the standard SSS methods for suppressing magnetic interference (Taulu and Kajola, 2005, Taulu and Simola, 2006). The cross-validation SSS method in the present study required more computing steps (see Appendix A for details). Altogether, the xSSS pre-processing time of a 15-min long recording was about 20 min on a 16GB RAM four-core Linux workstation (HP Z600). Creating the approximately 2400 beamformer virtual sensors took about 3 h on the same workstation.

### 3.3 Ripple detection

The ripple detection algorithm processed batches of 100 virtual sensors with 15 min of signal in 45 min on an 8GB RAM, 2.6 GHz CPU laptop. Detecting ripples in all 2400 channels per patient took about 18 h. It identified ripples in all patients before visual review, on average 905 ripples per patient (range: 79–3924). The review set consisted of 11 to 546 ripples per patient (average 148), and it took approximately 5 min per patient to review this set. The number of ripples excluded after visual review varied from 67 to 2950 per patient (average 737). The ripple detection algorithm thus had a false positive rate of 81.5%. This high false positive rate was accepted to ensure a good sensitivity. The majority of false positive detections were movement artefacts or EMG-like activity.

### 3.4 Ripple rates

After reviewing, 16 of the 25 patients (64%) showed ripples. In these 16 patients, on average 18 ripple-times were identified, which were on average 261 ripples per patient, with an average rate of 1.31 per minute (Table 2). Ripples were found on 165 virtual sensors on average, and this number was not correlated to the total number of virtual sensors in a patient (Spearman's rho(23) = 0.36, *p* = 0.08).

**Table 2:** MEG results: number of virtual sensors (VS) in each patient, duration of the recording, location of the identified ripples, concordance between ripples and MEG spikes, concordance between ripples and the resection area, the number of moments that a ripple was found in at least one channel (ripple-times), the total number of VS that showed ripples, the ripple-times per minute, and the concordance between MEG spikes and the resection area. Concordance is classified as good (+), moderate (=) or bad (−).

Pt #	VS	Duration (min)	Location MEG ripples	Concordance ripples with MEG spike dipoles	Concordance ripples with resection	Ripple-times	VS with ripples	Rate/min	Concordance spikes with resection
1	2649	14.8	L frontal + occipital	=		24	167	1.62	
2	2447	14.8	N			0	0		+
3	2480	15.3	Bilateral frontal + some widespread	=		42	816	2.75	
4	2384	15.1	R central	+	+	6	36	0.40	+
5	2224	14.4	N			0	0		−
6	2061	10.0	R centro-temporal	+		2	21	0.20	
7	2326	18.3	N			0	0		
8	2454	15.6	N			0	0		=
9	2275	8.6	R temporo-occipital	+	+	1	22	0.12	+
10	2363	17.1	N			0	0		=
11	2227	15.0	R frontal + L temporal	=	−	8	55	0.53	=
12	2269	15.0	R frontal + R fronto-central	+		15	112	1.00	
13	2768	9.6	R > L parieto-occipital	+	=	29	368	3.02	=
14	2436	14.6	L fronto-temporal + R occipital	−		3	4	0.21	
15	2778	15.0	R temporal	+	+	13	97	0.87	+
16	2313	14.5	R temporal posterior	+	=	4	12	0.28	+
17	2321	15.0	R temporal posterior	−		1	4	0.07	
18	2060	14.7	N			0	0		
19	2450	15.2	Bilateral frontal	+		12	260	0.79	
20	2364	5.7	N			0	0		−
21	2463	13.6	R parieto-occipital + some widespread	+		109	358	8.01	
22	2788	16.5	R parietal	+	=	16	285	0.97	=
23	2564	15.2	N			0	0		+
24	2393	15.0	R central + L temporal + R occipital	=	−	2	19	0.13	−
25	2663	15.0	N			0	0		+

*L: left, R: right, N: none*.

### 3.5 Ripple locations

**Fig. 5:**
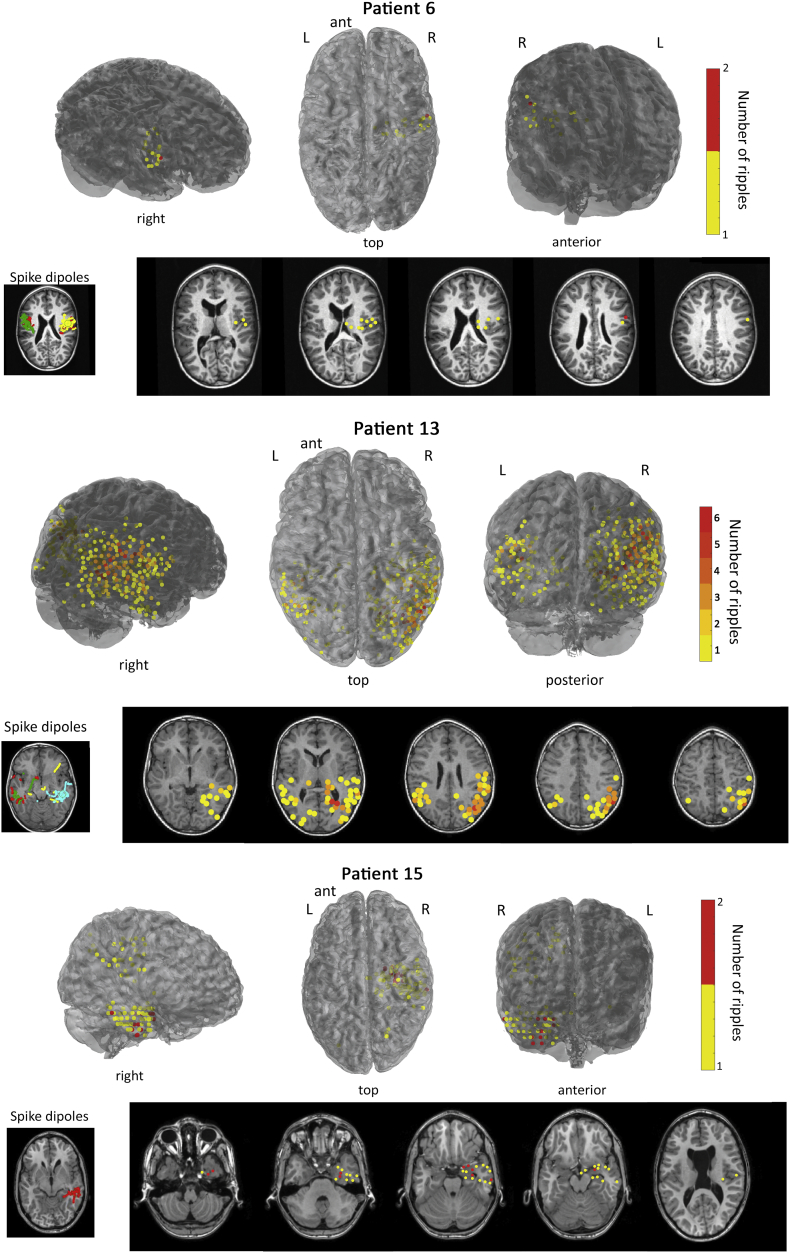
Ripple results for three patients. Ripples are visualized in a 3D figure (top), as well as axial MRI slices (bottom right). The ripple locations are compared to the spike dipoles from the clinical report (bottom left). All three patients show good concordance with MEG spike dipoles, as all ripple locations are also spike locations at lobar level. For patient 6, ripples were found unilaterally right centro-temporal, and spike dipoles were fitted bilateral centro-temporal. This was classified as good concordance, as the ripple location was also a spike location. Patient 6 did not undergo surgery because the number of seizures was too low. Patient 13 underwent surgery where a cortical tuber right frontal and a tuber right temporal were removed, but the seizure frequency did not change (Engel 4B). Patient 15 underwent a right temporo-lobectomy with amygdalohippocampectomy and was seizure free (Engel 1A). Postoperative MRI was not available.

Visual analysis showed good concordance of the location of the ripples at the lobar level with the location of the MEG spikes in 10/16 patients with ripples. Four patients showed moderate concordance, because some ripple locations were outside of spike locations. Of these four patients, the main focus of ripples in two patients (1 and 3) was also a spike location. Bad concordance was seen in two patients (14 and 17), both with only few ripple-times (3 and 1) and few channels with ripples (4 channels both, Table 2). Examples for individual patients are shown in Fig. 5.

Eight patients with ripples underwent surgery, of whom five were seizure free after resection (Engel score 1). Patient 4 and 15 were seizure free, and the MEG ripples showed good concordance with the resection site. The other three patients who were seizure free showed a moderate (patient 16 and 22) or bad (patient 24) concordance between MEG ripples and the resection site. In all three a temporo-lobectomy with amygdalohippocampectomy was part of the surgery. The three patients with ripples who did not become seizure free showed good (patient 9), moderate (patient 13) and bad (patient 11) concordance with the resection site. Patient 9 had an incomplete resection of the lesion. The MEG spikes in patients 11 and 13 were multifocal, and did not perform better than ripples in identification of the resection site.

We also determined the concordance between the MEG spike dipole locations and the resection area. For the eight patients with ripples who underwent surgery the spike and the ripple concordance were the same in six patients, and the spikes performed better than the ripples in the other two patients. For all ten patients who underwent surgery with good outcome, the spikes showed good concordance with the resection site in six patients, moderate concordance in 2 patients and bad concordance in 2 patients (Table 2).

### 3.6 Comparison with visual analysis

**Fig. 6:**
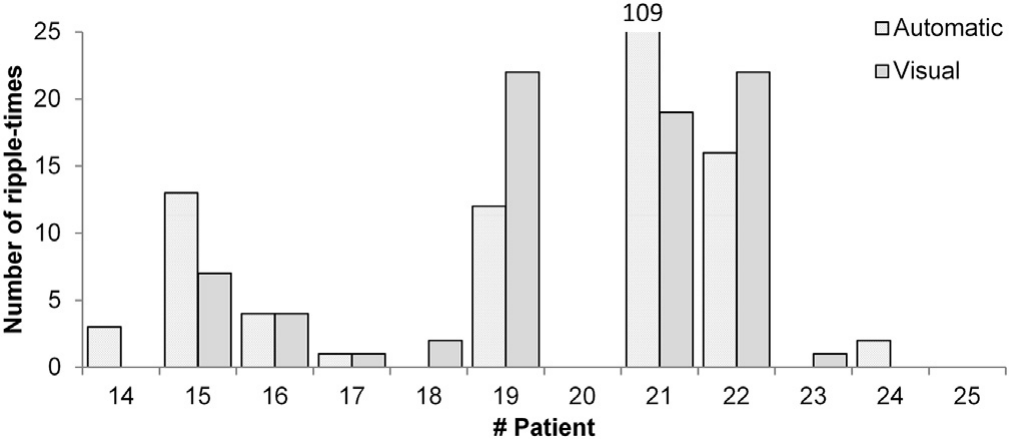
Number of automatically identified ripple-times compared to the number of visually marked ripples in (van Klink et al., 2016a). The numbers are comparable and not significantly different (*p* = 0.78).

The number of ripple-times identified by automatic and visual analysis were comparable and not significantly different (Wilcoxon Signed Rank Test, Z = − 0.28, *p* = 0.78, Fig. 6). Only for patient 21 the difference was striking, as we found 109 ripple-times automatically, and only 19 by visual marking. This is probably due to the limited spatial sampling of the visually marked sensors.

Ripples were marked visually in 8/12 patients; in 6 of whom ripples were also found automatically. The two patients in whom visually marked ripples were not detected automatically had only 1 and 2 visual ripple-times. Two patients in whom we did not find ripples visually, showed ripples after automatic detection.

## 4 Discussion

We show the feasibility of automatic detection and visualization of ripples in clinical MEG recordings. We used cross-validation SSS pre-processing and beamformer virtual sensors to increase the SNR and therefore were able to find ripples in 16 of the 25 patients in this study (64%). We validated these ripples by comparison with MEG spike dipole findings, which showed good or moderate concordance in 14 of the 16 patients with ripples. For six out of eight patients who had surgery, the ripple locations showed good or moderate concordance with the resection area: 4/5 with good outcome and 2/3 with poor outcome. Performing this analysis required only minimal review of the detected ripples, allowing for application in clinical practice.

The large amount of data of MEG routinely acquired in pre-surgical assessments requires a good data analysis strategy. The approach has to be accurate, as well as fast and easy to use for non-specialists, to be useful in clinical practice. Automatic detection algorithms for ripples usually have a high false positive rate, to ensure all true ripples are caught (Zelmann et al., 2012). This is especially crucial in MEG, where the ripple-rates are very low compared to intracranial recordings (Von Ellenrieder et al., 2016, Van Klink et al., 2016a). Visual review of the automatically detected events is usually the solution, but even this is a cumbersome job when > 300 channels with 80 Hz high pass filtered signal have to be reviewed. Our proposed algorithm takes time to run – approximately 3 h to create 2400 virtual sensor signals and 18 h to run the ripple detector on all these channels – but these steps are unsupervised. Determining the virtual sensor locations can also be automated. By creating a smart subset of detected ripples to review visually, the time a reviewer needs to spend on ripple analysis in one patient is reduced to 5 min to check the subset of detected ripples and exclude the false detections. The complete procedure, from raw MEG data to detected ripples, took approximately 21.5 h per patient, in which maximum half an hour of human work is involved, to initially check the quality of the recording and to check the subset of detected ripples.

The fact that ripples can be found in non-invasive MEG and EEG was long considered impossible, because the generators would be too small (Von Ellenrieder et al., 2014). The number of studies disproving this statement is growing, especially in EEG. The high density of MEG sensors and the ease to create a forward model for MEG would suggest that MEG is more suitable for HFO analysis than clinical EEG. However the magnitude of the background noise in MEG, and the interference induced by electrical power lines, vehicles, or heart beats, for example, might deem this untrue (Vrba, 2002). Passive or active shielding, smart geometry of gradiometers and magnetometers, synthetic higher order gradiometers (Vrba, 2002), signal space separation (Taulu and Kajola, 2005, Taulu and Hari, 2009), and beamforming (Hillebrand et al., 2016, Van Klink et al., 2016a) can be used to improve the SNR. In a previous study we have shown that it is possible to identify epileptic ripples in the time domain in MEG data that was pre-processed (Van Klink et al., 2016a). In that study we only sampled a small area of interest, and calculated beamformer virtual sensors based on spike markings. In this study we used the whole 80 Hz filtered 15 min signal as data covariance, thus minimizing the effect of a small covariance matrix on the quality of reconstructed sources and power estimation (Brookes et al., 2008). We further improved the SNR by using the cross-validation signal space separation (xSSS; manuscript in preparation) that reduced both magnetic interference and sensor noise. The resulting signals were of such quality that automatic detection of ripples was possible.

The rate of true ripples was 1.3/minute in patients with ripples, which is low, but comparable to visually marked ripples (Van Klink et al., 2016b). One other study that automatically detected ripples in the time domain found ripples in 8 out of 17 patients (47%), without using beamformer virtual sensors, and found similar ripple rates (Von Ellenrieder et al., 2016).

Analysis of ripples can be difficult, as filtering of sharp transients can result in ripple-like oscillations (Bénar et al., 2010). Filter artefacts of sharp transients show activity over all frequencies, low to high. Our automatic detector discards such high frequency activity that is part of broadband activity, because the power spectral density will not show a distinct high frequency peak (Fig. 2E). These ripples are considered artefacts. However, we often see ripples at the same time as epileptogenic spikes, which are not connected in the frequency spectrum, and therefore no filter artefacts. These were events that we considered as true ripples. Ripples in invasive recordings can also be a physiological phenomenon, and distinction between physiological and pathological ripples is difficult when no tasks are performed. Physiological ripples have not (yet) been found in spontaneous non-invasive recordings. As most ripples in our study seemed to relate to the epileptic focus, we assumed they were pathological.

With our proposed method, all moments a ripple was present in at least one channel (ripple-times) were considered. This resulted in a large area that seems involved in ripple generation. This is in contrast with the idea that ripples in intracranial ECoG are thought to be generated by only a small brain area (Jacobs et al., 2012). Reasons for the relatively widespread ripples in MEG can be found in the spatial smoothness of the measured magnetic fields (Ahonen et al., 1993), and the blurring effect, or leakage, of the spatial filtering beamformer algorithm (Barnes and Hillebrand, 2003, Hillebrand and Barnes, 2011). The diameter of the ripple cluster seemed also larger than the spike clouds, but dipole clouds were the result of analysis of several selected spikes, fitted over multiple latencies, and presented here without confidence volumes, while ripples were detected on each virtual channel independently. For these reasons, determining the actual size of the ripple generating area and comparison with spikes is difficult. To be able to draw conclusions on the ripple generation area, we would at least need to reconstruct the sources of spikes and ripples in a similar fashion.

The results for ripples as marker for the resection area were not better than those for spikes, and spikes were a good marker. Ripples should not replace spikes as a biomarker, but they can be an addition to the spike information, to strengthen the result of the MEG. Of course the added value of ripples would be larger if we can also find them in patients without spikes in MEG.

We used these spikes as gold standard to determine the reliability of the detected ripples, which is not the best gold standard, but one that was available for all patients. The best gold standard for identification of the epileptogenic zone is seizure freedom after resective surgery. As MEG in our centre is used mainly for patients without a clear hypothesis about the epileptogenic zone, i.e. the most difficult cases, unfortunately only eight patients with ripples were considered eligible for epilepsy surgery, of which 5 were successful. The resection area was concordant with the MEG ripples in two of them. Interestingly, the other three patients had a temporo-lobectomy with amygdalohippocampectomy, which suggests that detection of deep mesiotemporal sources is difficult. The insensitivity of MEG to sources in the mesiotemporal lobe has been stated before (Hillebrand and Barnes, 2002), also for ripples (Von Ellenrieder et al., 2016). However, even for two of these difficult cases we still found moderate concordance, suggesting that the improved SNR offered by beamforming (and xSSS in this study) may come to our aid, as shown previously for interictal spikes (Hillebrand et al., 2016). In the patients with poor outcome after surgery, ripples showed concordance with the resection area in two of the three patients, which can indicate that the resection was incomplete.

We included patients with spikes in the unfiltered MEG. The presence of spikes was not required to perform the analysis, but it increased the chance of finding ripples (Melani et al., 2013). We found ripples in 64% of the patients with spikes in the MEG, which is in line with 61–88% of focal epilepsy patients with ripples that are reported in scalp EEG (Andrade-Valenca et al., 2011, Melani et al., 2013, Van Klink et al., 2016b). Our results also suggest that the chance of good localization is higher when the number of identified ripples is higher. Therefore the performance of the method might improve when longer epochs are analysed.

We used a method to facilitate ripple detection in the time domain, where the virtual electrode time series were constructed on the basis of the whole recording. The localization of the detected ripples could be improved by applying source localization in the ripple band at the ripple-times, combining information from all channels at all ripple-times, and thereby increases the SNR in the spatial and temporal domain. This would also give more insight in the true size of the ripple generating area. Methods such as beamforming (Hillebrand et al., 2005) or the wavelet maximum entropy of the mean approach (wMEM, (Lina et al., 2014)) could be used for such a next step to improve localization accuracy of the automatically identified ripples.

## 5 Conclusion

We generated beamformer virtual sensors throughout the brain to increase the chance of finding ripples, and detected these ripples with an automatic detection algorithm with minimum human intervention. We have shown that this approach is feasible and that the identified ripples correlated with the MEG spike dipoles and with the resected area in the subset of patients who were successfully operated. Further validation of the MEG ripples as a biomarker for the epileptogenic zone has to be performed in a larger cohort of patients who underwent surgery. This automatic analysis method paves the way for such studies.

## Funding

N. van Klink is supported by the Dutch Brain Foundation fund (number 2013-139) and the Dutch Epilepsy Foundation fund 15-09. S. Burnos is supported by Vontobel Stiftung, EMDO Stiftung, Herzog-Egli Stiftung and Swiss National Science Foundation (SNSF 320030_156029). S. Taulu is supported by a grant from the Washington State Life Sciences Discovery Fund (LSDF). The Wellcome Trust Laboratory for MEG Studies at the Aston Brain Centre, Aston University, UK, is supported by the Wellcome Trust and the Dr Hadwen Trust for Humane Research. M. Zijlmans is supported by the Rudolf Magnus Institute Talent Fellowship 2012 and ZonMW Veni 91615149. J. Nenonen, L. Helle and S. Taulu are or were employed by Elekta Oy. There are no further potential conflicts of interest to be disclosed.

## Appendix A

### A.1 The cross-validation SSS method

Signal Space Separation (SSS) (Taulu and Kajola, 2005) is a method for processing multichannel magnetic recordings on the basis of quasistatic Maxwell's equations and the geometry of the measurement. In MEG, SSS has been applied for suppressing external interference (software shielding), compensation for signal distortions caused by head movements, and normalization of head positions (Taulu and Kajola, 2005). The spatiotemporal tSSS method based on temporal correlations removes also nearby interference (Taulu and Hari, 2009, Taulu and Simola, 2006). The cross-validation SSS (xSSS) method has two important novelties: cross-validation for extracting and suppressing uncorrelated channel artefacts and noise, and covariance-based regularization of the SSS reconstruction for reducing numerical reconstruction noise (manuscript in preparation; conference abstract has been presented by (Taulu et al., 2012)).

SSS reconstructions may suffer from artefacts or noise that are unique to a specific channel, as is the case when, for example, a channel is malfunctioning. Such artefacts may affect the accuracy of the SSS model and consequently leak to other channels. The cross-validation algorithm leaves out MEG channels one by one, and reconstructs, using SSS, the channel signal using the data from all other channels. The reconstructed signal is an accurate representation of the true signal, assuming perfect calibration accuracy and an overdetermined system, i.e., a multichannel recording with the number of channels exceeding the number of degrees of freedom. Even if the calibration information is not accurate, which affects the accuracy of the SSS model, the reconstructed channel signal based on cross-validation is still completely free of sensor noise and artefacts corresponding to the channel under investigation. Thus, the difference between the measured and reconstructed signal contains the signal component that does not have any correlation with the signals of other channels. The approach provides two important benefits: 1) It detects channels with large artefacts, e.g. due to a malfunctioning sensor, and neglects such channels in subsequent SSS reconstructions. 2) After the application of cross-validation, each channel signal is free of its own noise and artefact contribution that was originally uncorrelated with all other channels. The method, however, is not able to fully suppress spatial spreading of the uncorrelated sensor noise and artefacts to other channels, but the overall noise level will be reduced by cross-validation.

The spatial SSS transforms measured *N*-channel MEG signals *b* into *M* device-independent harmonic function amplitudes *x* (*M* < *N*):

b=Sx,

where the matrix ***S*** contains the harmonic basis functions (Taulu and Kajola, 2005). Instead of estimating the amplitudes *x* with the pseudo-inverse,

$$x={\left(S^{T}S\right)}^{-1}S^{T}\;b,$$

information of the signal and noise covariance matrices (***C***_x_ and ***C***_n_) provides efficient regularization for the estimates (Foster, 1961):

$$x=C_{x}S^{T}{\left({\mathrm{SC}}_{x}S^{T}+C_{n}\right)}^{-1}\;b.$$

The details of the covariance estimation are presented elsewhere (manuscript in preparation). After covariance-based estimation of *x*, the SSS-reconstructed signals have lower sensor noise over the whole frequency band.

The xSSS method can also be combined with temporal estimation and reduction of disturbance waveforms caused by nearby interference. The disturbing waveforms are estimated as in tSSS (Taulu and Simola, 2006), but here they are projected out from the original signals *b* before any SSS operations. The cross-validation and covariance-based regularization are performed thereafter as described above.

### A.2 Pre-processing pipeline

We used a research software module (Elekta MaxFilter version 3.0, not commercially available) for pre-processing the data in four steps:

1) Computation of head positions and subtraction of the signal from the head localization coils, but no SSS operations (Uutela et al., 2001).
2) Identification of temporal waveforms caused by nearby interference sources, and time-domain projection of them from the data without doing SSS transform. We used a buffer length of 10 s and sub-space correlation of 0.9.
3) Cross-validation based identification of channels exhibiting large uncorrelated artefacts or excessive noise, for neglecting such channels in SSS operations.
4) SSS reconstruction using sensor-level cross-validation to subtract uncorrelated sensor artefacts and noise, and application of noise and signal covariance information in the SSS transform to reduce the sensor noise in the whole frequency band.

The steps could be performed in a single script which processed the whole pipeline automatically. We took a two-step approach by checking for channels with artefacts after step 3. The whole pre-processing of a 15-min long MEG recording took about 20 min on a four-core Linux workstation with 16GB RAM.
